# Electromechanical Deformations and Bifurcations in Soft Dielectrics: A Review

**DOI:** 10.3390/ma17071499

**Published:** 2024-03-26

**Authors:** Yipin Su, Xudong Shen, Zinan Zhao, Bin Wu, Weiqiu Chen

**Affiliations:** 1 Qihang Union & Innovation Center, Huanjiang Laboratory, Zhuji 311800, China; zhaozn@zju.edu.cn; 2 Department of Engineering Mechanics, Zhejiang University, Hangzhou 310027, China; bin.wu@zju.edu.cn; 3 Center for Soft Machines and Smart Devices, Huanjiang Laboratory, Zhuji 311800, China; vibration@zju.edu.cn

**Keywords:** dielectric elastomers, electromechanical coupling, bifurcation, electroelasticity

## Abstract

Dielectric elastomers have attracted considerable attention both from academia and industry alike over the last two decades due to their superior mechanical properties. In parallel, research on the mechanical properties of dielectrics has been steadily advancing, including the theoretical, experimental, and numerical aspects. It has been recognized that the electromechanical coupling property of dielectric materials can be utilized to drive deformations in functional devices in a more controllable and intelligent manner. This paper reviews recent advances in the theory of dielectrics, with specific attention focused on the theory proposed by Dorfmann and Ogden. Additionally, we provide examples illustrating the application of this theory to analyze the electromechanical deformations and the associated bifurcations in soft dielectrics. We compared the bifurcations in elastic and dielectric materials and found that only compressive bifurcation modes exist in elastic structures, whereas both compressive and tensile modes coexist in dielectric structures. We summarize two proposed ways to suppress and prevent the tensile bifurcations in dielectric materials. We hope that this literature survey will foster further advancements in the field of the electroelastic theory of soft dielectrics.

## 1. Introduction

Electroactive materials are a category of smart materials known for their remarkable ability to respond to electrical stimuli by altering their physical properties [1,2]. This electromechanical coupling characteristic provides a smart, convenient, and controllable means for regulating device performance, making electroactive materials ideal candidates for smart devices and systems. Piezoelectric materials are typical representatives of electroelastic materials and find widespread applications at the core of modern technologies, such as surface acoustic wave devices, ultrasonics, sonar, sensors, and transducers [3,4]. The discovery of piezoelectric materials can be dated back to 1880 when French physicists Pierre Curie and his brother Paul-Jacques Curie observed the piezoelectric effect: placing a heavy object on a quartz crystal resulted in the generation of electric charges on the crystal’s surface [5].

In the subsequent years, an increasing number of functional devices based on piezoelectric materials have been developed, and research on the mechanical properties of piezoelectric materials has become more refined and comprehensive [6,7]. However, piezoelectric materials can be relatively brittle, making them prone to mechanical failure under excessive stress or impact [8]. Moreover, the production of high-quality piezoelectric materials can be costly due to the specialized manufacturing processes required [9]. These limitations pose clear challenges in developing high-performance smart devices.

The last two decades have witnessed an explosion of the applications of soft dielectric elastomers (DEs), which can undergo reversible and large deformation with a fast response to voltage. In addition to electromechanical coupling, DEs are soft and biocompatible, which points to a promising future in areas such as bioengineering, contact lenses, biosensors, and drug-delivery systems [10,11]. Compared to piezoelectrics, DEs exhibit superior performance in actuation strain, reaction speed, fracture toughness, drive power, etc. (for example, see Table 1 from [12]). Taking these advantages of DEs, a large number of novel types of smart devices have been manufactured.

It has long been recognized that theoretical research can provide references for guiding the design and fabrication of actuators [13]. The complex multi-field coupling and nonlinear characteristics of DEs have posed numerous problems and challenges in the study of their mechanical properties, and there is an urgent need to develop new theoretical, experimental, and computational methods. The nonlinear theory of electroelasticity was originally proposed by Toupin for the analysis of static motion in DEs [14]. Over the following decades, mechanical and engineering researchers have made significant efforts in unifying electromagnetic principles with classical elasticity theory [15,16]. In recent years, with the widespread application of DEs capable of undergoing significant deformation, there has been a renewed interpretation and development of nonlinear electorelastic theory [17,18,19]. At this stage, this theory was extensively used in the study of various mechanical properties of the DE materials and structures, including nonlinear response [20,21,22], vibration [23,24,25,26,27], wave motion [28,29,30,31], instability [32,33,34], Photonics [35,36,37], etc.

Several efforts have been devoted to summarizing the advances in the study of DEs from different aspects. For example, a systematic review was conducted by Zhao et al. on the current research regarding the vibration and wave characteristics of DEs, highlighting the shortcomings in the present studies [38]. Lu et al. summarized the application of the nonlinear electroelastic theory for solving the boundary-value problems in DE structures with varying configurations and provided a review of the performance of existing DE transducers [39]. Zhu et al. reviewed some recent theoretical and numerical efforts in exploring the mechanical properties of DEs, with emphases on the governing equations of electromechanical coupling, constitutive laws, viscoelastic behaviors, electromechanical instability, as well as actuation applications [40].

The present survey intends to summarize the recent advances in the study of nonlinear responses and the associated bifurcations in DE structures and provide prospects for future research work in these areas. In Section 2, we first briefly review the nonlinear theory of electroelasticity and the associated linear incremental theory proposed by Dorfmann and Ogden. Then, we revisit the existing works on the nonlinear responses and bifurcations in DE elastomers and structures, respectively, in Section 3 and Section 4. Finally, in Section 5, we draw some conclusions and outlooks.

## 2. Basic Formulations

Here, we choose Dorfmann and Ogden’s theory [17,28] as a typical representation to introduce the theory of nonlinear electroelasticity, as it combines the classic nonlinear elastic theory with electromagnetics, presenting a unified framework for nonlinear electromechanical theory, which is easy to follow and has been extensively applied in the analyses of DE materials and structures. In fact, there exist quite different versions of the nonlinear field theory for a deformable solid with electromechanical coupling. Interested readers may refer to the review article by Wu et al. [41] for further information, where the authors conducted a comparative analysis of different electroelastic theories developed in recent years, and clarified the relationships among them.

The theory of electroelasticity in the literature generally includes two parts: one is the nonlinear field theory describing the large-deformation stage, which can be used for solving the boundary-value problems of DE materials and structures under different static loading and boundary conditions; the other part concerns the incremental motions (which can either be small or large) superimposed on the finite deformation, called incremental theory, which can be used for the study of the dynamic problems in DEs, including wave propagation, vibration, bifurcation, etc. Theoretically, the incremental theory can be derived by expanding the corresponding constitutive laws, governing equations, and boundary conditions obtained in the first part into the Taylor series. Typically, the DEs are often taken to be incompressible, because the change in the shape of the material is much larger than that in the volume [42]. Thus, here, we are just concerned with the electroelastic theory for incompressible DEs, but note that both the theories for incompressible and compressible DEs are presented in Dorfmann and Ogden’s works [17].

### 2.1. Finite Electroelasticity

Consider an initially undeformed homogeneous electrostatic solid. It deforms from the reference configuration into the current configuration when subject to a combined action of mechanical and electric loads. The constitutive equations of the body read

(1) $$T=\frac{\partial \Omega }{\partial F}-pF^{-1},\;E_{l}=\frac{\partial \Omega }{\partial D_{l}},$$

where $T=F^{-1}\tau$ is the nominal stress in the body, with τ being the total Cauchy stress tensor, $\Omega =\Omega \left(F,D_{l}\right)$ is the energy function of the material defined in the reference configuration, F=∂x/∂X is the deformation gradient tensor, with X and x representing the material particle in the reference and current configurations, respectively, *p* is a Lagrange multiplier associated with the incompressibility constraint of the material, which can be determined from the governing equations and boundary conditions, $E_{l}=F^{T}E$ is the nominal electric field, with E representing the true electric field, and $D_{l}=F^{-1}D$ is the nominal electric displacement, with D representing the true electric displacement.

In the absence of body forces, free charges, and currents, the equilibrium equations in the solid read

(2) $$\mathrm{div}\tau =\rho x_{,tt},\;\mathrm{curl}E=0,\;\mathrm{div}D=0,$$

where ρ is the mass density of the material in the current configuration and *t* is time.

We assume that there is no surface charge, and the boundary conditions are

(3) $$\tau n=t_{a}+\tau^{*}n,\;\left(E-E^{*}\right)\times n=0,\;\left(D-D^{*}\right)\cdot n=0,$$

where n is the outward unit normal vector of the boundary in the current configuration, $t_{a}$ is the applied mechanical traction per unit area of the boundary of the solid in the current configuration, $\tau^{*}=\varepsilon_{0}\left[E^{*}⊗E^{*}-\frac{1}{2}\left(E^{*}\cdot E^{*}\right)I\right]$ is the Maxwell stress, with $\varepsilon_{0}$ = 8.85 pF/m being the permittivity of the vacuum, and $E^{*}$ and $D^{*}$ are the electric field and electric displacement exterior to the body, respectively. We note that the relationships $D^{*}=\varepsilon_{0}E^{*}$, $\mathrm{curl}E^{*}=0$, and $\mathrm{div}D^{*}=0$ always hold in vacuum. Equation ${\left(3\right)}_{1}$ and Equation ${\left(3\right)}_{2,3}$ are known as the mechanical and electrical boundary conditions, respectively.

The nonlinear response of the elastomer can be determined by solving Equations (1)–(3), once the energy function Ω, initial deformation F, and boundary conditions are given. Three widely used energy functions are

(4) $$\Omega_{G}=-\frac{\mu J_{m}}{2}\ln\left(1-\frac{I_{1}-3}{J_{m}}\right)-\frac{\varepsilon }{2}I_{5}$$

for ideal Gent DE elastomers [43],

(5) $$\Omega_{\mathrm{nH}}=\frac{\mu }{2}\left(I_{1}-3\right)-\frac{\varepsilon }{2}I_{5}$$

for ideal neo-Hookean DE elastomers [44], and

(6) $$\Omega_{O}=\sum_{n=1}^{N}\frac{\mu_{n}}{\alpha_{n}}\left(\lambda_{1}^{\alpha_{n}}+\lambda_{2}^{\alpha_{n}}+\lambda_{3}^{\alpha_{n}}-3\right)-\frac{\varepsilon }{2}I_{5}$$

for ideal Ogden DE elastomers [45]. Here, $I_{1}$ and $I_{5}$ are two of the five principal invariants, $\lambda_{1},\;\lambda_{2}$, and $\lambda_{3}$ are the principal stretches of the deformation, *N* is a positive integer, $\mu =\sum_{n=1}^{N}\mu_{n}\alpha_{n}/2$ is the initial shear modulus in the undeformed configuration, with $\mu_{n}$ and $\alpha_{n}$ being the material constants, $J_{m}$ is the nondimensional stiffening parameter, and ε is the permittivity of the solids. We note that, for the limiting case $J_{m}\to \infty$, the Gent model will degrade into the neo-Hookean model.

### 2.2. Incremental Field Theory

Next, we consider the incremental motions superimposed on the finitely deformed configuration. As mentioned above, the incremental theory can be obtained by expanding the governing equations, i.e., Equations (1)–(3), into the Taylor series. Throughout the paper, we are concerned with the bifurcations in DE materials and structures, which can be determined by using the linearized theory of infinitesimal incremental fields. Thus, here, we only retain the first-order terms in the Taylor expansion. Hereinafter, dotted variables represent incremental quantities.

By expanding Equations (1)–(3), we can obtain the incremental constitutive equations as

(7) $${\dot{T}}_{0}=A_{0}H+\Gamma_{0}{\dot{D}}_{l0}+pH-\dot{p}I,\;{\dot{E}}_{l0}=\Gamma_{0}^{T}H+K_{0}{\dot{D}}_{l0},$$

the incremental equilibrium equations as

(8) $$\mathrm{div}{\dot{T}}_{0}=\rho u_{,tt},\;\mathrm{curl}{\dot{E}}_{l0}=0,\;\mathrm{div}{\dot{D}}_{l0}=0,$$

and the incremental boundary conditions as

(9) $$\begin{array}{cc} \; & {\dot{T}}_{0}^{⊤}n={\dot{t}}_{A0}+{\dot{\tau }}^{*}n-\tau^{*}H^{⊤}n+\left(\mathrm{div}u\right)\tau^{*}n,\\ \; & \left({\dot{E}}_{10}-{\dot{E}}^{*}-H^{⊤}E^{*}\right)\times n=0,\\ \; & \left[{\dot{D}}_{10}+HD^{*}-{\dot{D}}^{*}-\left(\mathrm{div}u\right)D^{*}\right]\cdot n=0, \end{array}$$

respectively, where ${\dot{T}}_{0}$, ${\dot{D}}_{l0}$, and ${\dot{E}}_{l0}$ are the ‘push forward’ versions of the incremental nominal stress, nominal electric displacement, and nominal electric field, respectively, $A_{0}$, $\Gamma_{0}$, and $K_{0}$ are fourth-, third-, and second-order electroelastic moduli tensors, which are determined once the finite deformation is given, and H=gradu is the displacement gradient, with $u\left(x,t\right)=\dot{x}\left(X,t\right)$ being the incremental displacement.

## 3. Nonlinear Deformations in DE Structures

One of the most intriguing characteristics of DE materials and structures is their ability to undergo significant deformation. A wealth of existing literature was dedicated to studying large deformations in DE materials and structures, including those with plate, membrane, cylindrical, and spherical configurations.

### 3.1. Plate

Compared to other configurations, plates have the advantages of a simple structure, the ease of mechanical modeling and solving, and convenience in manufacturing. Therefore, they are often used to elucidate the principles of dielectric actuation and the characteristics of electromechanical coupling. The working principle of the DE plate is that, in the presence of an electric field, large electrostrictive stresses can be generated to drive the deformation of the elastomer. For a plate with its surface free of traction, but subject to in-plane mechanical loadings $P_{1}$ and $P_{2}$ along the $x_{1}$ and $x_{2}$ directions and a nominal electric $E_{0}$ along the thickness, it generates attractive forces in the DEs that lead to a decrease in the actuator’s thickness and an increase in its surface area. The DE plate will deform according to the mapping [46]:

(10) $$x_{1}=\lambda_{1}X_{1},\;x_{2}=\lambda_{2}X_{2},\;x_{3}=\lambda_{1}^{-1}\lambda_{2}^{-1}X_{3},$$

where $\lambda_{i}\;\left(i=1,2\right)$ is the principal stretch along the $x_{i}$ direction. Then, the deformation gradient of the solid can be written as $F=\mathrm{diag}\left[\lambda_{1},\lambda_{2},\lambda_{1}^{-1}\lambda_{2}^{-1}\right]$.

Generally, there are two different loading methods to activate the DEs: the voltage-control method and the charge-control method.

#### 3.1.1. Voltage Control

**Figure 1:**
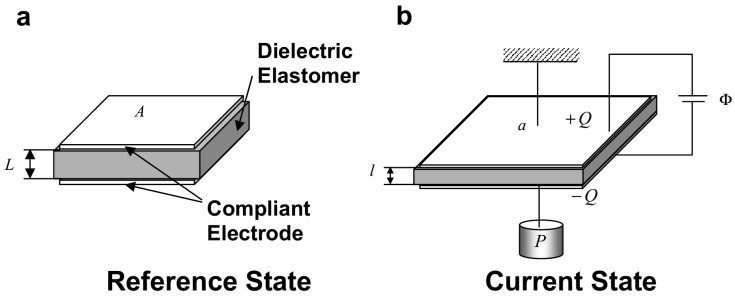
A DE plate subject to equibiaxial stretch with a voltage normal to the major surfaces: (**a**) reference configuration; (**b**) current configuration (reproduced from [47] with permission).

As shown in Figure 1, a standard voltage-controlled DE actuator with high deformability usually consists of a soft elastomer sandwiched between two electrodes [47]. The electrode can be typically composed of conductive carbon grease [48] or graphite powder [49], for example. It is flexible and can be considered to have no effect on the deformation of the elastomer. Typically, the plate expands in the area and thins down homogeneously when subject to a voltage through the thickness. However, Zhao and Suo [50] proposed a thermodynamic model to show that the electric field in the solid may cause DEs to become thinner or thicker, depending on the deformation-dependent permittivity of the material. Plante and Dubowsky [51] experimentally revealed that this homogeneous deformation can become unstable, leading to the emergence of an inhomogeneous deformation. In this scenario, two different kinds of regions coexist in the layer: one remains flat, while the other develops wrinkles. Zhao et al. [47] developed a modeling framework to demonstrate how a homogeneous deformation in the DE layer can lead to the emergence of two coexistent states. The results showed that the free-energy function typically exhibits nonconvex behavior, resulting in the elastomer undergoing a discontinuous transition from a thick state to a thin state. When both states coexist within the elastomer, the thin state exhibits large areas and wrinkles when constrained by nearby regions of the thick state.

For DEs undergoing equibiaxial deformation ($\lambda_{1}=\lambda_{2}$), the material will experience the so-called electromechanical instability (also known as snap-through or pull-in instabilities). In the work of Zhao and Suo [44], the authors theoretically studied an equibiaxially stretched DE plate. It was shown that the applied voltage induces the in-plane stretches of the plate, which further increases as the voltage increases. The peak of the mentioned curve they obtained is identified when the Hessian matrix, also known as the generalized tangent modulus, loses its positive definiteness, representing the occurrence of the electromechanical instability of the elastomer. Further increasing the voltage will induce a sudden and considerable plate expansion, leading to the risk of electrical breakdown. They also showed that the applied in-plane prestress can markedly increase the actuation stretch. We note that the authors conducted numerical calculations for ideal DEs, whose dielectric permittivity is independent of the deformation of the material, although the theory proposed was valid for materials with any form of energy function. Afterward, some researchers conducted extensive work to extend this theory to other energy functions. For example, Liu et al. [52] considered the permittivity of the material as a variable of the deformation in the free-energy function and showed that an appropriate elastic strain energy function of a specific DE material can be used to predict the electromechanical stability. Norris [53] examined the electromechanical stability of ideal Ogden DEs and presented a comparison with experimental data in the literature [51,54]. Liu et al. [55] studied the electromechanical stability of ideal Mooney–Rivlin DEs and obtained the analytical expression of the Hessian matrix.

The electromechanical coupling property of DEs leads to multiple failure characteristics that are not presented in elastic materials, requiring in-depth research into their competitive and conversion mechanisms. By assuming that the free energy of the DE elastomer is simply the addition of polarizing and stretching energies, Díaz-Calleja et al. [56] studied the competition between electric and mechanical force fields simultaneously applied to a DE elastomer that can lead to electric breakdown. Zhao and Wang [57] categorized the deformation and instabilities of soft DEs into three main types: (i) thinning and pull-in, (ii) electro-creasing, leading to cratering, and (iii) electro-cavitation. They offered a comprehensive analysis of these different deformation and instability modes in soft DEs, combining cutting-edge experimental techniques, theoretical models, and practical applications, and discussed a range of methods to either mitigate or exploit these deformations and instabilities in soft DEs for a variety of applications. It has been experimentally observed that the attainable deformation of actuation in different types of DE materials varies markedly [58,59,60,61]. In order to investigate the fundamental factors that limit the deformation, Zhao and Suo [62] proposed the theory of DEs capable of achieving giant deformation of actuation. By comparing the critical values for the onset and stop of snap-through instability with those for electrical breakdown, they divided DEs into three categories: type I DEs, whose electrical breakdown occurs before the onset of snap-through instability, type II DEs, whose snap-through instability will induce electrical breakdown of the material, and type III DEs, which survive the pull-in snap-through instability without electrical breakdown. Clearly, type III DEs are the most ideal materials due to their significantly larger maximal actuation strain compared to types I and II. Su et al. [63] investigated the effects of material stretchability and electrostriction on the snap-through instability of an incompressible Gent DE plate and found that material constants can be carefully chosen to design type III DEs.

#### 3.1.2. Charge Control

**Figure 2:**
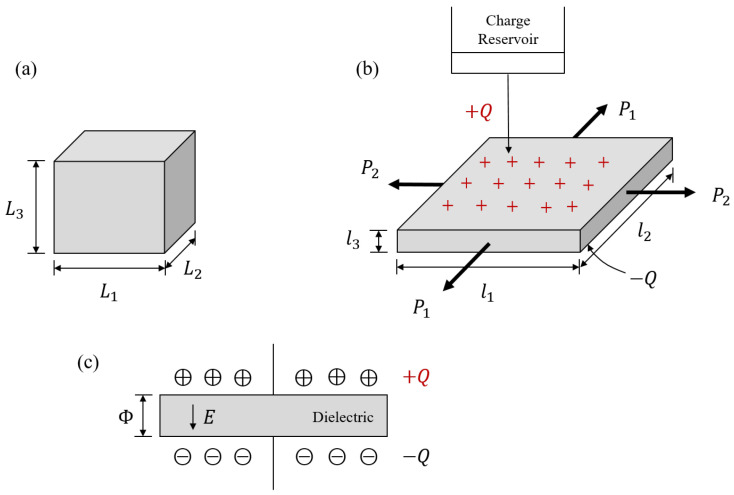
A DE plate with sprayed charges on its bottom and top faces and subject to in-plane stresses: (**a**) reference configuration; (**b**) current configuration; (**c**) the electric field in the solid is induced by the charges (reproduced from [64] with permission).

As depicted in Figure 2 [64], the electric field in DEs to activate a deformable DE capacitor can be generated by spraying charges on the top and bottom surfaces, with [65] or without [66] electrodes.

Lu et al. [65] studied electromechanical localization and electrical breakdown in a charge-controlled DE plate. They observed that, when electric charges remain stationary on the surfaces, charge-controlled actuation remains stable and avoids pull-in instability. Conversely, in the presence of electrodes, charges become mobile, rendering charge-controlled actuation bistable. Upon reaching a critical charge, uniform deformation becomes unstable, causing the capacitor to transition abruptly to a state of localized deformation through a first-order transition, potentially resulting in electrical breakdown. Li et al. [64] proposed a thermodynamic framework to characterize electromechanical stability in charge-controlled actuation, with a particular focus on the electrostatic energy generated by constant charges and its interaction with deformation. They investigated the electrostatic stress caused by these charges and compared it with Maxwell stress. Their findings showed that charge-controlled deformation remains stable without the pull-in instability, due to the decrease in electrostatic stress with stretch. Su et al. [67] established criteria for identifying pull-in instability in DEs activated by various methods, including voltage control, charge control, fixed pre-stress, and fixed pre-stretch, by analyzing the free energy in the actuated systems. Numerical calculations were performed for ideal neo-Hookean DEs to determine the maximum actuation stretch a DE can sustain before electrical breakdown. They discovered that applying a fixed pre-stress or pre-stretch to charge-driven DEs may reduce their stretchability, which contradicts the behavior observed in voltage-driven DEs.

### 3.2. Membrane

DE membrane/film capacitors can be manufactured using reactive magnetron sputtering [68]. They have found extensive applications in microelectronics, power electronics, power grids, medical devices, etc., due to their outstanding mechanical advantages. These advantages include a broad range of capacitance, high-voltage operation, ultra-fast charge and discharge capability, minimal raw resource consumption, lightweight modules, and graceful failure reliability [69,70,71,72,73].

To the best knowledge of the authors, the pioneering work on nonlinear deformations in DE membranes was conducted by Goulbourne et al. [20]. They proposed a comprehensive mathematical formulation that encompasses large deformations, material nonlinearity, and electrical effects to accurately model DE membranes. This formulation utilizes Maxwell–Faraday electrostatics and nonlinear elasticity. The resulting analytical model was employed to numerically investigate the behavior of an inflatable DE membrane, considering variations in system parameters such as prestrain, external pressure, applied voltage, and the percentage of electrode-covered membrane area. This model can be used to predict acceptable ranges of motion based on specified system specifications. The effectiveness and correctness of the theory were validated by comparing theoretical predictions with existing experiments [74] in fluid pump applications.

**Figure 3:**
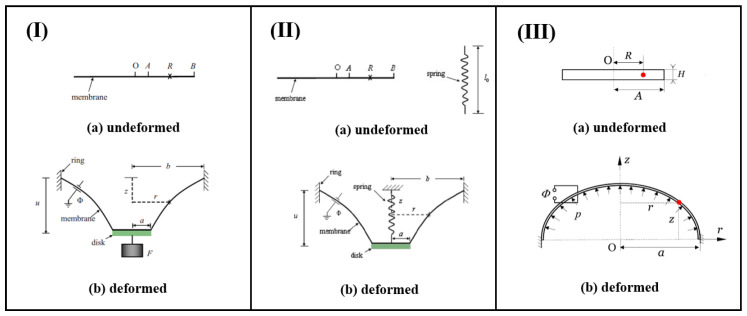
A circular DE membrane undergoes non-uniform out-of-plane deformation subject to a voltage through the thickness and a vertical mechanical force. (**I**) The vertical force is induced by an attached weight (reproduced from [75] with permission); (**II**) The vertical force is induced by an attached spring (reproduced from [77] with permission); (**III**) The vertical force is induced by an internal pressure. Here the red dot represents any point at a distance *R* from the center of the membrane at the undeformed configuration (reproduced from [79] with permission). As a result, the solids response from (**a**) the undeformed configuration to (**b**) the deformed configuration.

DE membranes undergoing non-uniform out-of-plane deformation are a classic configuration used to elucidate the material’s electromechanical coupling deformation mechanism. To induce such a deformation in the material, an out-of-plane mechanical force needs to be applied. For example, applying a voltage while simultaneously attaching a rigid circular disk to a DE membrane will result in non-uniform deformation (Figure 3I. He et al. [75] theoretically studied a DE membrane deformed into an out-of-plane axisymmetric form. They derived the governing equations for the inhomogeneous state of equilibrium, considering deformation, charging kinematics, and thermodynamics. Their numerical results indicated that the electric field within the membrane can exhibit significant inhomogeneity, and the membrane is prone to various failure modes, including electrical breakdown, loss of tension, and rupture due to excessive stretching. Later, this work was extended by Wang et al. [76] to consider the time-dependent dissipative behaviors of the material.

Unlike suspending heavy objects, He et al. [77] also examined the static non-uniform out-of-plane deformation of a DE membrane. In this case, the out-of-plane mechanical force is provided by a vertical spring (Figure 3II. They formulated the fundamental equations governing significant out-of-plane deformations and performed numerical solutions of these equations. They showed that the anticipated displacement of the disk can be controlled by adjusting either or both of the parameters of the spring and the applied voltage. Wang [78] also examined the same configuration, but considered the influence of material viscoelasticity. Both the creep behavior under constant instantaneous electro-mechanical loads and the cyclic performance under cyclic mechanical and electrical loads were studied.

In addition, applying internal pressure can also lead to non-uniform out-of-plane deformation of the DE membranes (Figure 3III). The viscoelastic deformation of a DE membrane mounted on a rigid circular ring and subjected to a combination of pressure and voltage was studied by Wang et al. [79]. They used a nonlinear spring–dashpot model to capture the viscoelastic behavior of the material. The governing equations consisted of a series of first-order ordinary differential equations and initial and boundary conditions, which can be solved using the shooting method. The authors conducted numerical calculations for ideal neo-Hookean materials. It was observed that, when small pressures and voltages are applied, the DE membrane gradually transitions to an equilibrium state. However, when high pressures and voltages are applied, the membrane fails to reach equilibrium, potentially leading to electromechanical instability over time.

### 3.3. Solid and Hollow Tubes

As one of the widely used configurations, tubular DE actuators have a very low inactive-to-active-material ratio and are less bulky [80,81], making them less cumbersome and more adaptable for various applications. One potential use for them is in the manufacturing of large-volume pumps [82,83].

#### 3.3.1. Inflation and Extension

For an incompressible DE tube undergoing inflation and extension, the material deforms follows the mapping [29]:

(11) $$r=\sqrt{\lambda_{z}^{-1}\left(R^{2}-R_{i}^{2}\right)+r_{i}^{2}},\;\theta =\Theta ,\;z=\lambda_{z}Z,$$

where (R,Θ,Z) and (r,θ,z) are cylindrical coordinate systems for the undeformed and deformed configurations, respectively, $\lambda_{z}$ are the principle stretches in the axial direction, and $R_{i}$ and $r_{i}$ are the initial and deformed inner radii, respectively. Then the deformation gradient can be determined as $F=\mathrm{diag}\left[\lambda^{-1}\lambda_{z}^{-1},\lambda ,\lambda_{z}\lambda_{2}^{-1}\right]$, with λ=r/R being the circumferential stretch of the tube.

**Figure 4:**
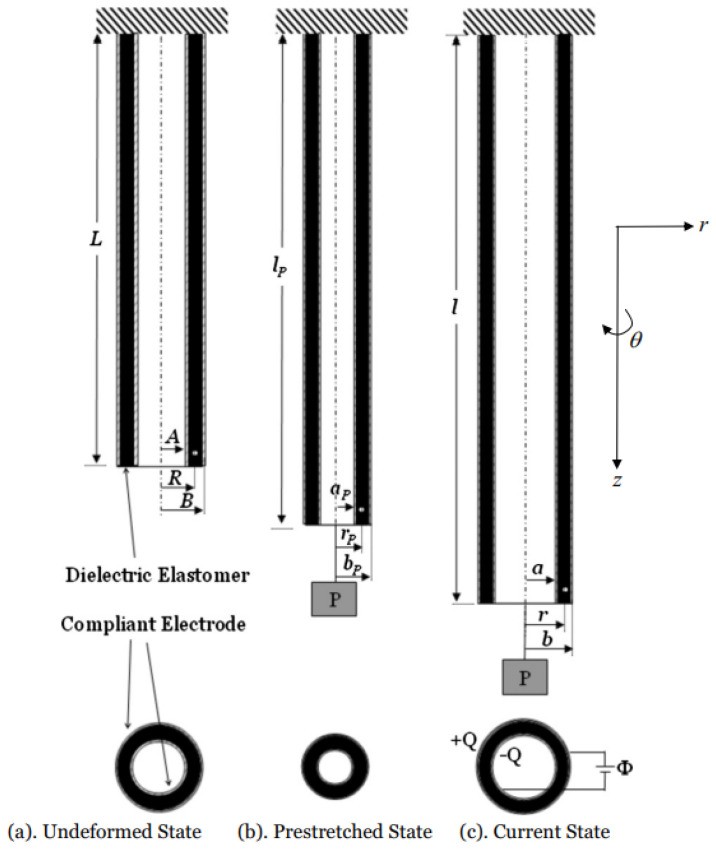
Diagram illustrating a dielectric elastomer tube with two flexible electrodes on its inner and outer faces: (**a**) initially, the tube has a length *L*, with inner and outer radii *A* and *B*, respectively, in its unstretched form; (**b**) when pre-stretched by a force *P*, its length becomes $l_{p}$, and the inner and outer radii adjust to $a_{p}$ and $b_{p}$, respectively; (**c**) under the influence of both a force *P* and an applied voltage, the tube reaches a length *l*, with inner and outer radii *a* and *b*, respectively (reproduced from [84] with permission).

Zhu et al. [84] conducted a theoretical examination of a DE tube actuator (Figure 4). They considered the ideal neo-Hookean model and obtained an analytical solution for the actuator’s behavior under significant deformation. It was shown that, when a voltage difference is applied across its inner and outer surfaces, the actuator’s thickness decreases, while its length increases, leading to an increase in the electric field, which may result in electric breakdown. He and Wang [85] proposed a model incorporating energy dissipation using advanced viscoelastic electroelastic theory [86] to examine the electro-viscoelastic behavior of a tubular DE actuator constructed by forming a DE membrane into a cylindrical shape and securing it with rigid disks at both ends. When activated by internal pressure and voltage, the tube expands and transforms into a shape that extends beyond its original plane, experiencing substantial deformation. To describe this deformation, the authors utilized non-equilibrium thermodynamics to formulate state and governing equations and derived the kinetic equations using a rheological spring–dashpot model. The results indicated that low pressure or voltage leads the actuator to a stable state, whereas high pressure or voltage results in instability, either mechanical or electromechanical. Su et al. [30,87] studied the deformation of DE tubes subject to an axial homogeneous electric field. It was shown that, in that case, the deformation is homogeneous throughout the tube. They presented a detailed investigation of the influences of the biasing fields, the electromechanical coupling parameters, and the geometrical parameters on the deformation of the solid. That work was then extended by Wu et al. [29] to examine the inhomogeneous deformations in DE tubes subject to a radial electric field. They considered the ideal neo-Hookean materials and derived the analytical expressions for the stresses and electric fields in the tube. Bortot [88] proposed theoretical modeling to explore the electromechanical behavior of multilayer DE tubes. The study presented numerical data demonstrating how the mechanical and geometrical characteristics of the soft coating layers affect the electromechanical performance of the active membrane under various constraint conditions. It was shown that electric loading results in a radial expansion in axially constrained tubes, axial extension and cavity expansion in radially constrained tubes, and both axial extension and expansion in unconstrained tubes.

#### 3.3.2. Torsion

For DE tubes undergoing torsion, the deformation is given by [89]

(12) $$R^{2}-R_{i}^{2}=\lambda_{z}\left(r^{2}-r_{i}^{2}\right),\;\theta =\Theta +\gamma \lambda_{z}Z,\;z=\lambda_{z}Z,$$

where γ is the torsion angle per unit length. Then, the deformation gradient reads

(13) $$F=\left[\begin{array}{ccc} \lambda^{-1}\lambda_{z}^{-1} & 0 & 0\\ 0 & \lambda & \gamma \lambda_{z}r\\ 0 & 0 & \lambda_{z} \end{array}\right].$$

**Figure 5:**
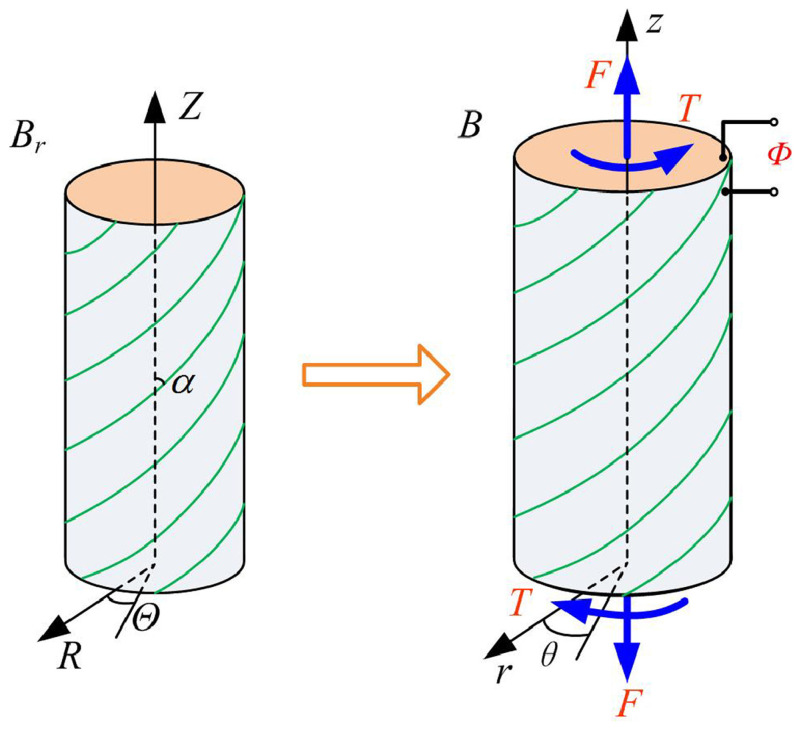
Illustrative diagram showing the initial (**left**) and current (**right**) configurations of a helical fiber-reinforced tubular DE actuator undergoing torsional deformation (reproduced from [90] with permission).

He et al. [90] analyzed the voltage-induced torsion and snap-through instability in a tubular DE actuator using an energy method (Figure 5). The research investigated how fiber stiffness, helical angle, and external mechanical loads influence the torsional deformation. The findings revealed that, at snap-through instability, the voltage-induced twist angle significantly increases from a smaller value in the unbulged state to a larger one in the bulged state, and this twist angle can be effectively adjusted by altering the fiber stiffness and helical angle. Furthermore, it was observed that external axial forces or torque has minimal effects on the actuator’s torsion in the bulged state. The finite axisymmetric torsion of a tubular DE actuator was analyzed by He et al. [91] using the membrane theory. A constitutive model for the fiber-reinforced DE actuator was developed by integrating the Mooney–Rivlin elasticity and ideal DE models, considering the inextensibility of the fibers. The authors examined how material properties, the fiber’s helical angle, and mechanical loads, such as internal pressure, axial force, and twist moment, influence the DE actuator’s voltage-induced torsional behavior. Bazaev and Cohen [92] explored a new design for a DE-based bi-layer tubular structure, functioning as an electrically activated torsional actuator. The bi-layer tube was constructed by twisting an isotropic Gent tube, inserting it into another tube, and adhering the two layers at their interface, creating residual stress and making the layers geometrically incompatible. When subject to an electric field, the bi-layer tube undergoes deformation-induced anisotropy, resulting in a twist. It was shown how the overall twist response can be manipulated by an appropriate choice of mechanical and electrical properties for the two layers. Su [89] studied the finite response of a DE tube subject to a combination of applied radial voltage, torsion, and axial force. They looked at illustrative numerical calculations for ideal Mooney–Rivlin DEs and explored how factors like applied voltage, mechanical stress, structural geometry, and actuation methods affect the finite response of the tube. The findings indicated that the deformation of the solid impacts the actual electric field within it, leading to a competitive interplay between the applied voltage and mechanical stress.

### 3.4. Spherical Balloons

A DE balloon is extensively utilized as a configuration for functional devices due to its ability to improve electric-induced deformation and its adaptability in various industrial applications [93]. Potential applications include high-frequency pumps [94,95] and loudspeakers [96,97], for example.

When subject to a radial voltage and an inner pressure, the DE balloon undergoes a spherically symmetric deformation described by

(14) $$r^{3}-a^{3}=R^{3}-A^{3},\;\theta =\Theta ,\;\varphi =\Psi ,$$

where (R,Θ,Ψ)and(r,θ,φ) are the spherical coordinate systems in the reference and current configurations, respectively, and *A* and *a* are the inner radii of the balloon in the reference and current configurations, respectively. Then, the deformation gradient of the solid reads $F=\mathrm{diag}\left[\lambda^{-2},\lambda ,\lambda \right]$, with λ=r/R being the principal stretch in the θ and φ directions.

**Figure 6:**
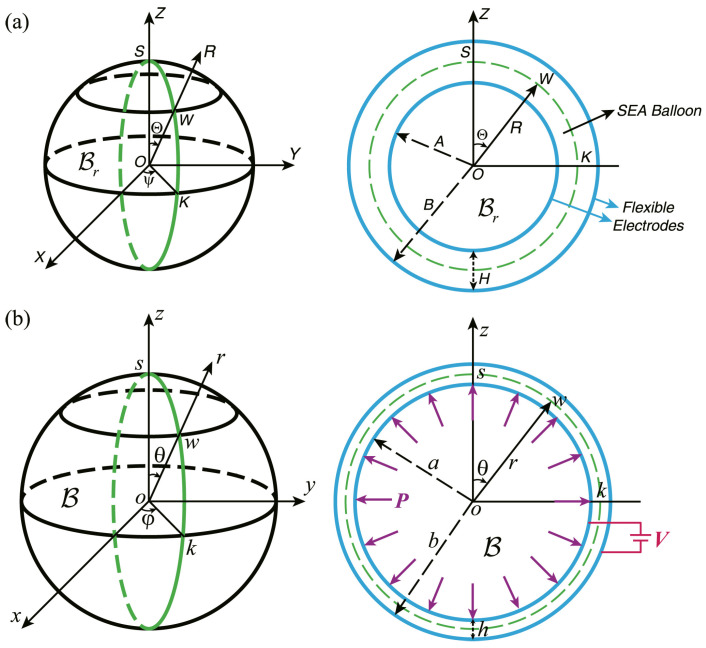
Illustrative representation of a DE balloon undergoing finite expansion when subject to a combination of a radial voltage and a mechanical inner pressure: (**a**) the reference and (**b**) the current configurations (reproduced from [99] with permission).

A solution was identified [98] for the issue involving DE spherical balloons subject to both mechanical and electrical stimulations, proposing the snap-through cycles for using these balloons as micro-pumps or actuators. The solution indicates that certain material responses may cause instabilities, resulting in abrupt changes in the size of the balloon. Based on the nonlinear theory of electroelasticity, Mao et al. [99] investigated the finite deformation of DE balloons, focusing on the influences of the internal pressure, radial electric voltage, and material and structural parameters on the deformation (Figure 6). They conducted calculations for DEs characterized by both neo-Hookean and ideal Gent DE models and found that the snap-through instability induced by both the electric voltage and the internal pressure can be exploited to realize sharp transitions in the balloon. Bortot [100] examined the electromechanical behavior of multilayer DE spherical balloons, which are either composed solely of the active membrane (single-layer balloon) or include a coated active membrane (multilayer balloon). Dorfmann and Ogden [45] analyzed radial deformations in a thick-walled spherical shell made of DE material, which has compliant electrodes on its inner and outer surfaces and is subject to internal pressure. They derived a general formula that relates the pressure to the deformation and either a potential difference between the electrodes or uniform surface charge distributions on them. For demonstrative purposes, specific forms of energy functions, including the neo-Hookean, Gent, and Ogden models, were utilized in numerical analyses for scenarios with either a fixed potential difference or a fixed charge distribution. Su et al. [34] explored the nonlinear deformation in layered DE balloons, where an elastic layer is attached outside the DE balloon. They developed a comprehensive mathematical model that is capable of explaining the finite inhomogeneous strains caused by electro-mechanical interactions in the solid. The numerical findings revealed the influence of the presence of the elastic layer outside the DE layer on the finite deformation of the balloon.

### 3.5. Finite Bending

**Figure 7:**
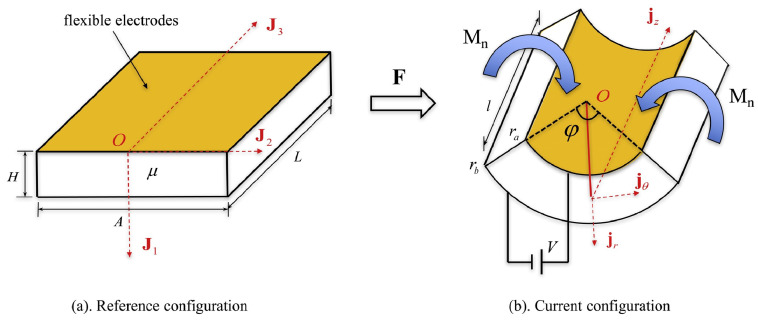
Sketch of a DE plate with a voltage applied across its thickness undergoing finite bending deformation: (**a**) the reference and (**b**) the current configurations (reproduced from [101] with permission).

Generally, moments are required to bend a DE plate, as depicted in Figure 7. The finite bending can be mathematically captured by the following mapping [101]:

(15) $$r=\sqrt{d+\frac{2X_{1}}{\omega }},\;\theta =\frac{\omega X_{2}}{\lambda_{z}},\;z=\lambda_{z}X_{3},$$

where $\left(X_{1},X_{2},X_{3}\right)$ and (r,θ,z) are the rectangular Cartesian and cylindrical coordinates in the reference and deformed configurations, respectively, *d* and ω are constants to be determined, and $\lambda_{z}$ is the axial principal stretch, which is taken to be prescribed. Then, the deformation gradient reads $F=\mathrm{diag}\left[\lambda^{-1}\lambda_{z}^{-1},\lambda ,\lambda_{z}\right]$, with $\lambda =wr/\lambda_{z}$ being the principal circumferential stretch.

Finite bending deformation frequently occurs in DE-based devices [102,103,104], yet theoretical analysis of this deformation in DE structures has received limited attention. He et al. [105] theoretically explored the finite bending deformation of a DE actuator induced by voltage, applying the nonlinear electroelasticity theory and assuming a pure bending deformation. Theoretical and numerical analyses showed that incorporating pre-stretch can decrease the necessary drive voltage for achieving specific bending deformations. Moreover, at higher levels of pre-stretch, there is an optimal thickness ratio dependent on the pre-stretch, which can further minimize the required drive voltage. Su et al. [101] developed a mathematical model for analyzing the finite bending deformation of an incompressible DE plate (Figure 7), which is valid for materials with an arbitrary form of energy function. They provided an explicit treatment of the boundary-value problem of the finite bending and derived closed-form expressions for the stresses and electric fields in the body. It was shown that mechanical moments are required on the lateral faces to drive the bending in the plate, and they call this scenario ‘mechanical bending’. Later, they proposed a dielectric–elastic bilayer [106] capable of large bending deformation, which they called ‘smart bending’ because this deformation can be activated in a controllable way by varying the electric field only, without mechanical loadings. They created the bilayer by gluing a stretched DE elastomer to an initially undeformed elastic elastomer. They theoretically studied the bending in the bilayer and revealed how the bending angle of the bilayer can be adjusted by varying the applied voltage. It should be noted that the circumferential stresses in the two elastomers are not continuous at the interface, potentially leading to sliding, exfoliation, or cracking during the bending deformation. In order to avoid the discontinuity of stress, Su and colleagues [107] proposed a structural model of a functionally graded DE plate, with the shear modulus and permittivity varying linearly across the thickness. When a voltage is applied across the thickness of the material, it causes a non-uniform deformation, which can lead to overall bending. They studied the voltage-induced bending response of the functionally graded DE plate and derived closed-form expressions for the stresses and electric field in the body.

## 4. Bifurcations in DE Structures

In practical terms, substantial actuation resulting from the pull-in instability is highly desirable in DE actuators to achieve significant changes in surface area. Nevertheless, as mentioned in the previous studies, it has been indicated that a drastic reduction in material thickness can lead to electrical breakdown damage. Apart from electrical breakdown, DEs are susceptible to various other types of failures compared to standard elastic materials, such as localized necking [108] and bulging [83], which pose significant challenges in the development of DE-based devices. The intricate interplay and competitive mechanisms among these failure modes necessitate a thorough comparison to assess their reliability.

### 4.1. Half-Space

To the best knowledge of the authors, the theoretical study of bifurcations in DEs based on the framework of electroelasticity was first proposed by Dorfmann and Ogden [109]. To demonstrate the incremental theory, the authors applied it to an electroelastic neo-Hookean material and examined the stability of a half-space under pure homogeneous deformation with an external electric field perpendicular to its surface. Their findings revealed that the stability significantly relies on the values of the electromechanical coupling parameters in the constitutive equation.

### 4.2. Membrane

**Figure 8:**
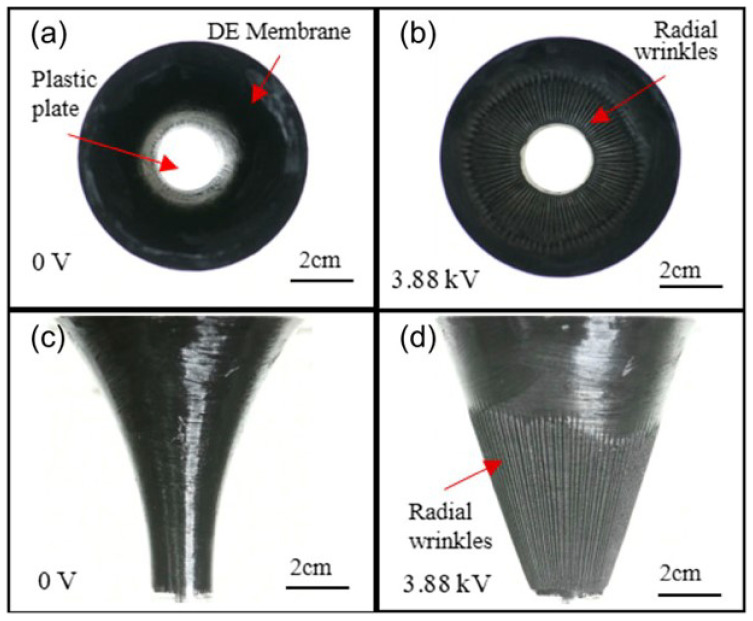
Wrinkling patterns in a trumpet-shaped sample when exposed to electrical voltage. Overhead perspectives (**a**,**b**) and lateral views (**c**,**d**) are presented both with and without the application of voltage (reproduced form the open-access article [111]).

Mao et al. [110] introduced a novel technique for producing regular (strip-like) and consistent wrinkles in a confined DE sheet, which is coated with soft electrodes on both sides and exposed to a high voltage. It was shown that, upon reaching a specific voltage threshold, negative stresses occur in the material, and wrinkles begin to form and expand. To explore the wrinkle’s wavelength and amplitude, the authors carried out both experimental and theoretical analyses. Their findings displayed a strong correlation between the theoretical predictions and experimental outcomes. This method was later expanded by Mao et al. [111] to create voltage-controlled radial wrinkles in a stretched circular DE membrane with a fixed boundary and to study the interplay between the wrinkle wavelength and the geometry of the circular membrane (Figure 8). They proposed a theoretical model and used it to derive an algebraic formula to predict the wrinkle wavelength. They found that the wrinkle wavelength of the trumpet-like DE membrane can be adjusted by modifying the displacement load, offering an advantage over previous designs with a fixed boundary.

### 4.3. Plate

**Figure 9:**
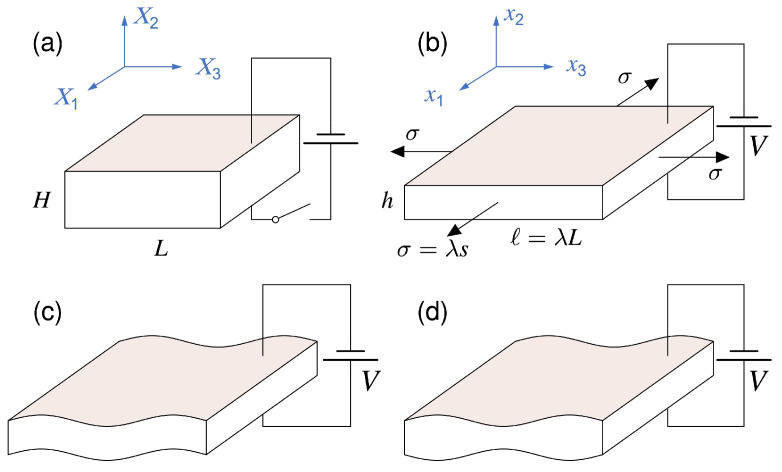
When subjected to a combination of electromechanical loadings, a soft DE plate uniformly deforms from (**a**) the reference configuration to (**b**) the current configuration. In cases of extreme deformation, the plate may lose its stability, leading to the formation of either (**c**) antisymmetric or (**d**) symmetric bifurcation modes (reproduced from [43] with permission).

As stated in the previous section, there are two typical methods to activate the DE actuators: voltage control and charge control. Dorfmann and Ogden [46] employed nonlinear electroelasticity theory to investigate the bifurcation modes of DE plates in these two scenarios: the first scenario involves generating an electric field using opposite charges on flexible electrodes attached to the plate’s main surfaces, while the second scenario involves an externally applied field without electrodes. In both cases, the primary finite deformation is associated with equibiaxial stretching, and the electric field is perpendicular to the plate’s major surfaces. They obtained the critical stretch at which the plate’s uniform initial configuration becomes unstable, which is expressed as a function of the plate’s initial thickness, electric field, and material model parameters. Numerical calculations were conducted for neo-Hookean ideal materials, and the results for the two cases were compared to show that the alternative option leads to essentially identical results. Motivated by the work of Ogden and Dorfmann, Díaz-Calleja [112] analyzed the bifurcation behaviors of DE plates made of Mooney–Rivlin and Ogden models, respectively. They showed that selecting more intricate models is essential to more accurately reflect the actual behavior of the solids. Yang et al. [113] examined the nonlinear response and bifurcation of ideal DE plates under plane-strain conditions. However, in the mathematical modeling, they focused solely on increments in the mechanical fields, disregarding changes in the electrical fields. Naturally, this simplification resulted in a more limited set of solutions, which is essentially a subset of the broader solution space that encompasses both incremental deformation and polarization. Fu et al. [108] developed a theory that captures the primary effects of thickness on the incremental bifurcation of a DE plate covered with electrodes. They expanded the bifurcation equation obtained by Dorfmann and Ogden [46] in a power series in the thickness direction for both the incremental deformation and electric field. Su et al. [43] revisited the finite expansion and bifurcation behaviors of voltage-controlled DE plates. They successfully derived the dispersion equation and decoupled it into explicit antisymmetric and symmetric modes (Figure 9). They obtained explicit formulas for the extreme scenarios of ultra-thin membranes and thick plates, which is valid for materials with arbitrary energy functions, and conducted numerical calculations for the neo-Hookean, Mooney–Rivlin, and Gent models, as well as DEs that exhibit polarization saturation. The results showed that, beyond the anticipated buckling mode seen in purely elastic scenarios, there exists a novel mode that emerges at high voltages during stretching. In the limit case of infinite thickness, the results obtained by this model were in perfect agreement with the solution Dorfmann and Ogden [109] obtained in a semi-infinite space. It was also observed that plates initially form wrinkles in an anti-symmetrical pattern before transitioning into symmetrical modes. The authors pointed out that the numerical results of Díaz-Calleja [112] do not reveal an extensional mode of wrinkling and seem to present numerical instabilities. Later, this work [43] was extended by Su et al. [114] to examine the effect of exterior electric fields. In the work by Dorfmann and Ogden [115], the Stroh method was developed for analyzing the bifurcation of DE plates. Unlike the previous works [43,46,113,114], the material constitutive law was given in terms of the energy function $\Omega^{*}\left(F,D_{L}\right)$. Explicit bifurcation equations were obtained for antisymmetric and symmetric modes of bifurcation, and the results were illustrated for a Gent electroelastic material model with different values of the Gent parameter.

Broderick et al. [116] investigated the bifurcation of a soft DE plate, which is deformed due to the combined action of in-plane mechanical pre-stresses and an electric field applied across its thickness under charge control. They demonstrated that charge-driven DEs, where the electric displacement varies linearly with the electric field, do not experience electromechanical instability. This type of instability, typically predicted by the Hessian criterion concerning the system’s free energy, is absent in charge-control scenarios.

### 4.4. Solid and Hollow Tubes

**Figure 10:**
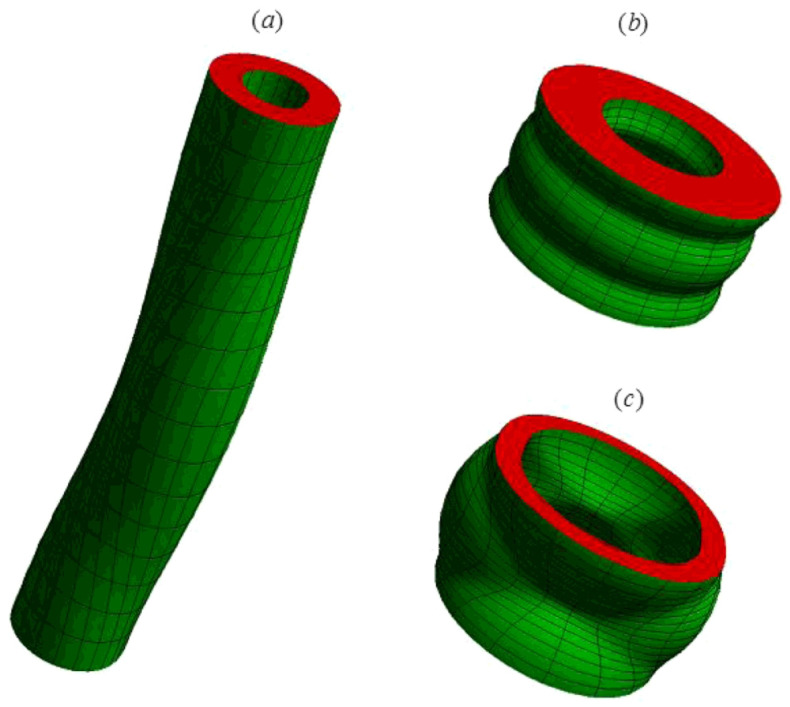
Bifurcation patterns of DE tubes with different material and structural parameters, where the red part represents the cross-section of the structure, while the green one represents the inner and outer surfaces: (**a**) a slender tube; (**b**) a short and thick tube; (**c**) a short and thin tube (reproduced from [87] with permission).

Su et al. [87] proposed a study on the bifurcation characteristics of incompressible DE tubes subject to homogeneous biasing fields, focusing on scenarios with and without external electric fields. They formulated and precisely solved the three-dimensional bifurcation equations in cylindrical coordinates using three displacement functions. The solution was articulated in terms of Bessel functions, and explicit frequency equations were provided for various scenarios. Numerical analysis revealed that the bifurcation patterns of the tubes can be tuned by several factors, including biasing fields, electromechanical coupling, cylinder geometry, and external electric fields (Figure 10). It should be noted that, for problems with homogeneous biasing fields, exact expressions of bifurcation equations can be derived, whereas for problems where the biasing fields are inhomogeneous, the differential system for the bifurcation equation is stiff, and advanced numerical methods should be employed. To solve the finite response and bifurcation of DE tubes subject to mechanical torsion and radial inhomogeneous electric field, Su [89] developed the amended surface impedance matrix method [117] to build a robust and efficient numerical procedure for obtaining the dispersion equation. It was revealed that electrically stimulated DE tubes may exhibit bifurcation in extension, as well as the anticipated contractile form, differing from standard elastic wrinkling. Additionally, introducing torsion enhances the electro-elastic properties of the DE elastomer. The results also showed that precise control of the actuation, voltage, torsion, and geometry enables the selection of specific wrinkling patterns in the tube, achieving substantial stable actuation without material failure. Melnikov et al. [118] conducted a bifurcation analysis of a circular cylindrical tube made of electroelastic material, featuring closed ends and flexible electrodes along its curved surfaces. They employed the so-called compound matrix method to build a robust numerical procedure for solving the resulting dispersion equations and determining the wrinkled shape of the tube at the onset of bifurcation. They performed numerical calculations for a neo-Hookean DE material and found that bifurcation modes can occur with inflation, in contrast to the purely elastic scenario, where prismatic bifurcations were only observed with external pressure. The tube is more prone to bifurcation in the presence of a moderate electric field. Bortot and Shmuel [119] explored a distinct category of bifurcations, termed ‘prismatic diffuse modes’, using the linearized theory of superimposed deformations on deformable DEs under finite strain. They formulated and solved governing equations to identify the emergence of these modes. Their numerical examples for ideal Gent materials demonstrated that the tube can transition into diffuse states before the failure of the Hessian criterion. Furthermore, they discovered that loading paths deemed stable by the Hessian method are capable of bifurcating into diffuse modes. Zhou [120] explored the combined effects of axial stretching and radial voltage on the finite deformation and bifurcation patterns of an incompressible, functionally graded dielectric tube. It was assumed that the elastomer’s modulus and permittivity linearly change across the tube’s thickness. They employed the surface impedance matrix method to derive the buckling bifurcation equation. Numerical analyses were conducted on an ideal neo-Hookean DE, examining the influence of applied voltage, geometric dimensions, and material gradation on the tube’s nonlinear and incremental buckling responses.

### 4.5. Spherical Balloon

**Figure 11:**
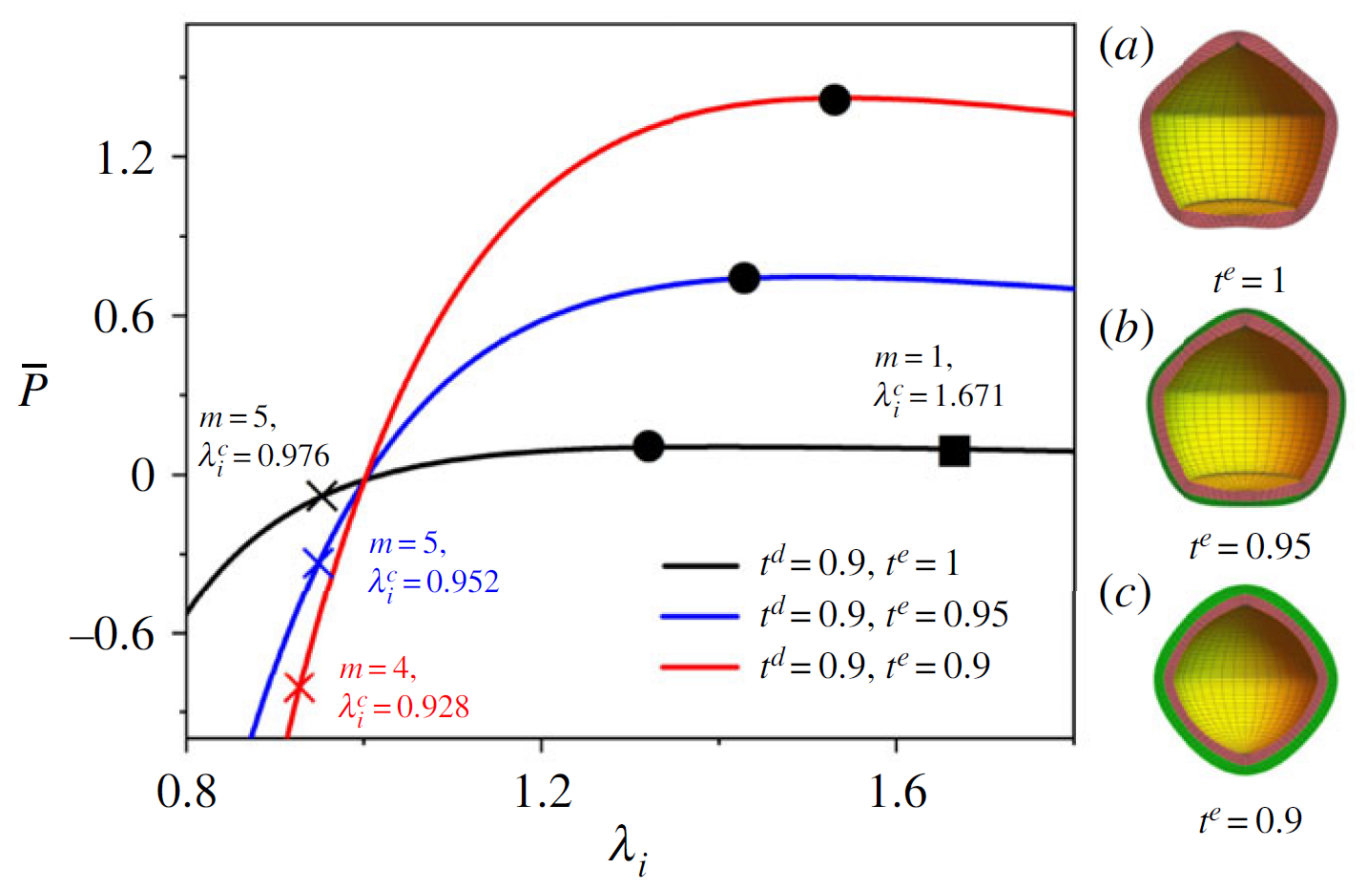
Inflation and bifurcation responses of pressure-activated dielectric–elastic balloons with varying thickness. The inner and outer layers are dielectric and elastic elastomers, respectively. The pressure-stretch curve is shown on the left, and the buckling shapes are shown on the right (**a**–**c**). The cross, circle, and square markers indicate the thresholds for bifurcation in compression, snap-through, and bifurcation in extension, respectively. It can be seen that the bifurcation modes of the structure can be designed by tuning the structural parameters of the balloon (reproduced from [34] with permission).

For an inflated elastic spherical balloon, it has been established that it can transform into a pear shape at the point where the tension in the membrane reaches a maximum, depending on the material energy function [121]. Later, this work was extended by Xie et al. [122] to examine the bifurcation of a DE balloon under pressurized inflation and electric actuation. The results indicated that, for a DE balloon, a transition into a pear shape is feasible for all material models, and it was revealed that, in cases where a pear-shaped form occurs, it consistently possesses a lower total energy compared to the simultaneous spherical configuration. Wang et al. [83] examined the unusual bulging patterns of a DE balloon under electromechanical load and theoretically derived the complete range of the DE balloon’s equilibrium states using thermodynamic principles. Stability analysis showed that anomalous bulging is typical for a DE balloon under electromechanical load, and various irregular shapes can be achieved through carefully planned loading paths. Notably, the areas of irregular bulging often experience the highest local strain, potentially leading to the DE membrane’s failure. Su et al. [34] delved into the complex deformation and the transitions between buckling, necking, and snap-through instabilities in layered DE balloons, triggered by an applied radial voltage and internal pressure. They explored the initial stages of morphological changes in spherically symmetrical balloons using the surface impedance matrix method and examined the nonlinear progression of the bifurcated states through finite-element numerical simulations. Their findings indicated the potential to create adjustable DE spheres, whose buckling and necking onsets can be manipulated through the geometric and mechanical characteristics of the passive elastic layers (Figure 11). It has long been established through experiments that the electromechanical responses of DEs are significantly dependent on the loading rate [123]. Mao et al. [124] proposed a theoretical analysis to explore how viscoelasticity influences wrinkle formation in a DE balloon subject to combined electromechanical loads. They conducted an experiment focused on the impact of viscoelasticity on wrinkle propagation in a DE balloon, and the experimental results strongly aligned with their model predictions. Melnikov et al. [118] studied the bifurcation behavior of a spherical DE balloon, which was pressurized and equipped with flexible electrodes on both the inner and outer surfaces. The analysis specifically examined axisymmetric bifurcations and presented findings for three distinct electroelastic energy models: those corresponding to neo-Hookean, Gent, and Ogden elastic energy functions. For the neo-Hookean model, it was previously established that axisymmetric bifurcations in a purely mechanical scenario occur only with external pressure, as internal pressure does not lead to bifurcations [125]. However, in an electroelastic neo-Hookean model, bifurcations can occur under internal pressure if the electrical potential difference across the electrodes surpasses a specific threshold, which is influenced by the ratio of the shell’s inner to outer undeformed radii.

### 4.6. Bending Bifurcations

**Figure 12:**
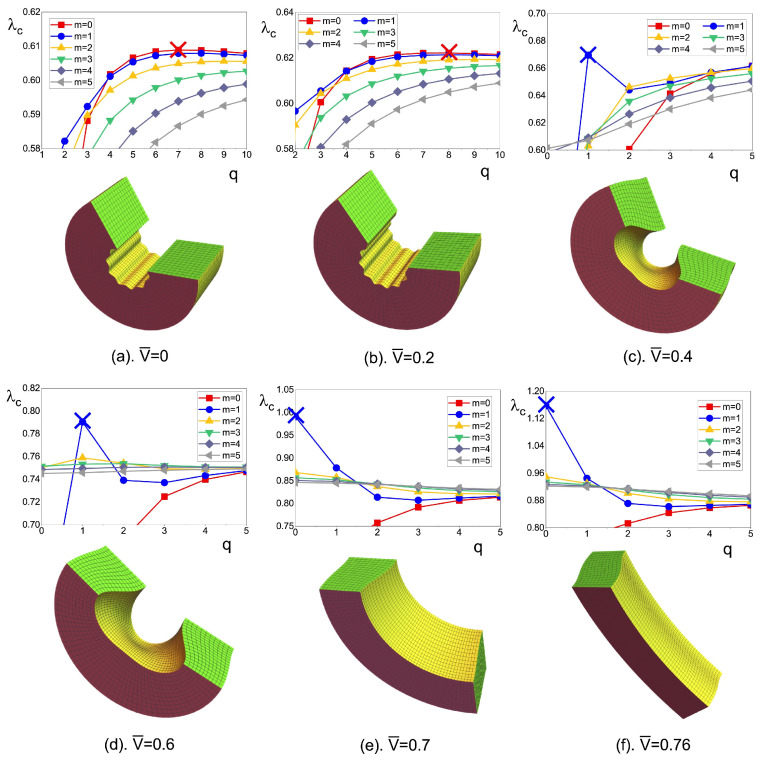
Patterns of DE slabs at the onsets of bending bifurcations: the top rows display the bifurcation curves representing various modes, while the bottom rows depict the wrinkling patterns that emerge during bifurcation. The red part represents the top and bottom surfaces, the green part represents the lateral surfaces, and the yellow part represents the inner and outer surfaces, respectively. In scenarios (**a**,**b**), circumferential wrinkles appear, while in cases (**e**,**f**), axial wrinkles become prominent. In contrast, cases (**c**,**d**) exhibit a two-dimensional pattern involving both circumferential and axial wrinkles (reproduced from [101] with permission).

As we have mentioned in Section 3.5, single DE plates for ‘mechanical bending’ actuation and dielectric–elastic bilayers for ‘smart bending’ actuation were proposed by Su and his colleagues [101,106]. In their works, the researchers have not only investigated the bending responses of DEs, but also proposed mathematical modeling on the associated bifurcation instability, which, to the best of the authors’ knowledge, were the only theoretical works on the bending bifurcation in DEs. In the work of mechanical bending of single DE plates, Su et al. [101] derived the analytical expressions of stresses and electric field, which is valid for DEs modeled by a general free-energy function. Illustrative numerical calculations were presented for ideal neo-Hookean DEs. It was shown that the applied voltage has a destabilizing effect on the bending instability of the slab, while the effect of the axial load is more complex: when the voltage is applied, changing the axial loading will influence the true electric field in the body and induce competitive effects between the circumferential instability due to the voltage and the axial instability due to the axial compression. The results indicated that the bifurcation shapes can be designed by tuning the voltage, material, and structural parameters of the DEs (Figure 12). Then, in their work on smart bending of dielectric–elastic bilayers, Su et al. [106] explored the limits of bending for the bilayer structure before it undergoes bifurcation instability when excessive compression is induced in the solid. Their findings indicated that the physical properties of both layers should be selected to be approximately equal in magnitude to achieve substantial bending without encountering bifurcation. If desired, the formation of wrinkles can be controlled to occur on either the inner or outer bent surface of the buckled bilayer.

## 5. Conclusions and Outlooks

### 5.1. Conclusions

The electromechanical coupling and nonlinear characteristics of DEs make the nonlinear deformation and bifurcation behaviors of these materials complex. Therefore, developing efficient computational models to address these challenges is essential. In this comprehensive review, we delved into the extensive body of research focusing on the nonlinear deformation and bifurcation instability of DEs. By investigating various DE configurations, researchers have made significant strides in understanding the complex interplay between material properties and nonlinearity.

Excessive deformation of DEs can lead to the occurrence of bifurcation instability. Unlike elastic materials, both tensile and compressive bifurcation modes coexist in DEs. It is worth noting that tensile instability can potentially lead to material failure, such as electrical breakdown, and needs to be avoided. Theoretically, it is possible to prevent the tensile instability mode in the material by applying pre-stretch, but this may also suppress snap-through deformation in the material [34]. To address this, researchers have proposed a method to simultaneously preserve snap-through deformation and suppress tensile instability: attaching an elastic layer to the exterior of the DE layer.

### 5.2. Outlooks

Despite significant progress in the study of nonlinear deformation and bifurcation stability in DEs, the authors believe that current research can still be improved in the following aspects:

1. Viscoelastic effects: As a unique class of soft materials, DEs exhibit significant viscoelastic characteristics. A nonlinear electromechanical coupling theory incorporating the viscoelastic characteristics has been proposed [86]. However, much of the existing research simplifies the modeling by overlooking this aspect. Accounting for the time-dependent mechanical response of DEs will deepen our comprehension of their behavior under diverse loading conditions and across prolonged durations.

2. Anisotropy: Anisotropy is another crucial factor deserving further attention in future studies [126]. Numerous DEs display directional dependencies in their mechanical properties, and comprehending the effects of anisotropy on nonlinear deformation and bifurcation will yield a more thorough understanding of their behavior across various scenarios.

3. Experimental validation: To validate theoretical and computational findings, future research should focus on rigorous experimental validation. Employing advanced testing methodologies and instrumentation will enhance the reliability and applicability of the insights gained from theoretical studies.

## Figures and Tables

**Table 1:** Comparison between different smart materials (reproduced from [12] with permission).

Type	Maximum Strain (%)	Maximum Pressure (MPa)	Specific Elastic Energy Density (J/g)	Relative Speed (Full Cycle)
dielectric elastomer (acrylic with prestrain)	380	7.2	3.4	medium
dielectric elastomer (silicone with prestrain)	63	3	0.75	fast
dielectric eastomer (Silicone—nominal prestrain)	32	1.36	0.22	fast
dielectric elastomer (polyurethane—nominal prestrain)	11	1.6	0.087	fast
electrostrictor polymer (P(VDF-TrFE-CFE))	4.5		1.1	fast
electrostatic devices (integrated force array)	50	0.03	0.0015	fast
electromagnetic (voice coil)	50	0.1	0.003	fast
piezoelectric ceramic (PZT)	0.2	110	0.013	fast
piezoelectric single crystal (PZT-PT)	1.7	131	0.13	fast
piezoelectric polymer (PVDF)	0.1	4.8	0.0013	fast
relaxor ferroelectric polymer (PVDF-TrFE-CFE)	7	21	0.73	fast
shape memory alloy (TiNi)	>5	>200	>15	slow
shape memory polymer (polyurethane)	100	4	2	slow
thermomechanical twisted polymer fiber (nylon-6,6 monofilament)	35	16		slow
thermal (expansion—Al dT = 500 K)	1	78	0.15	slow
conducting polymer (PANI)	0.85	100	0.32	slow
IPMC	3	30		
carbon naotubes (CNT paper)	1	27	0.04	slow-fast
magnetostrictive (terfenol-D)	0.2	70	0.025	fast
natural muscle (peaks in nature)	100	0.8	0.04	slow-fast
